# In vivo MR spectroscopy for breast cancer diagnosis

**DOI:** 10.1259/bjro.20180040

**Published:** 2019-07-02

**Authors:** Uma Sharma, Naranamangalam Raghunathan Jagannathan

**Affiliations:** Department of NMR & MRI Facility, All India Institute of Medical Sciences , New Delhi, India

## Abstract

Breast cancer is a significant health concern in females, worldwide. *In vivo* proton (^1^H) MR spectroscopy (MRS) has evolved as a non-invasive tool for diagnosis and for biochemical characterization of breast cancer. Water-to-fat ratio, fat and water fractions and choline containing compounds (tCho) have been identified as diagnostic biomarkers of malignancy. Detection of tCho in normal breast tissue of volunteers and in lactating females limits the use of tCho as a diagnostic marker. Technological developments like high-field scanners, multi channel coils, pulse sequences with water and fat suppression facilitated easy detection of tCho. Also, quantification of tCho and its cut-off for objective assessment of malignancy have been reported. Meta-analysis of *in vivo* ^1^H MRS studies have documented the pooled sensitivities and the specificities in the range of 71–74% and 78–88%, respectively. Inclusion of MRS has been shown to enhance the diagnostic specificity of MRI, however, detection of tCho in small sized lesions (≤1 cm) is challenging even at high magnetic fields. Potential of MRS in monitoring the effect of chemotherapy in breast cancer has also been reported. This review briefly presents the potential clinical role of *in vivo* ^1^H MRS in the diagnosis of breast cancer, its current status and future developments.

## Introduction

Breast cancer account for high morbidity and mortality in females throughout the world.^1^ Diagnosis of breast cancer at an early stage, *i.e*. when the tumor size is small is challenging. X-ray mammography has been used primarily for both routine screening and for detection of breast lesions, however, it has limitations in dense breast especially in young females.^2,3^ Ultrasound is specifically useful for the diagnosis of cysts, abscesses, lesions in dense breast and in guiding core needle biopsy; however, it has limitations in identifying microcalcifications. Significant overlap was reported in the morphology of benign and malignant lesions on ultrasound images.^2,3^ Conventional MRI and dynamic contrast-enhanced MRI (DCEMRI) have potential as an adjunct modality for diagnosis of mammographically occult, multifocal lesions, pre-operative tumor staging, tumor recurrence and in monitoring therapeutic response of the tumor.^4,5^

Over the last two decades, *in vivo* proton (^1^H) MR spectroscopy (MRS) has been shown to have the potential as a non-invasive tool for diagnosis and to provide an insight into the biochemistry of living tissues. MRS studies have reported raised water content^6^ and choline-containing compounds (tCho) in malignant breast lesions which were shown to discriminate them from benign lesions and enhance the diagnostic specificity of MRI.^7–21^ However, tCho has been observed in benign lesions as well as in normal breast tissues of volunteers and lactating females.^8,22–24^ These findings necessitated the development of *in vivo* quantification methods for tCho. Recently, several studies have reported the absolute concentration of tCho in malignant breast lesions^7,23–33^ and its cut-off value for the differentiation of malignant, benign and normal tissues.^23^ The association of tCho levels with hormonal receptor status and molecular markers like β-catenin has also been investigated.^34^ Monitoring tCho levels following chemotherapy to predict the response of the tumor to chemotherapy have also been reported.^8,11,35–40^ Thus, there has been considerable progress and promising results emerged from breast MRS in the last decade; however, breast MRS is still not a routinely used protocol in a clinical setting as in brain pathologies. Several technical factors, like complexity of acquisition procedures, optimization of analysis methods and patient comfort need to be addressed before its inclusion in a clinical setting. This article briefly highlights the methodology and reviews its applications in the diagnosis and assessment of breast cancer.

## Breast cancer metabolism and ^1^H MRS

The rapid and uncontrolled growth of cancer cells occurs due to dysregulation of various regulatory pathways leading to changes in several metabolic pathways.^41,42^ The malignant transformation thus results in alterations of the relative concentration of several cellular metabolites which can be measured by MRS. Changes in lipid and membrane metabolisms have received specific attention of breast MR specialists as it is possible to obtain quantitative measurements of metabolites like water, lipids and choline. Increased proliferative activity of malignant cells changes the cell membrane metabolism resulting in elevated tCho levels in the *in vivo* ^1^H MR spectrum of breast cancer.^7–33^ Several compounds including free choline, phosphocholine (PCho) and glycerophosphocholine (GPC) contribute to the tCho peak observed around 3.2 ppm in the ^1^H MRS. Choline containing compounds PCho and phosphoethanolamine (PE) are used as precursors through the Kennedy pathway for synthesis of phospholipids and phosphotidylcholine (PtdCho) which are subsequently utilized for the cell membrane synthesis. The PtdCho synthesis is regulated by activity of three enzymes choline kinase, CTP-cytidyl transferase and phosphocholine transferase. Briefly, choline is phosphorylated by choline kinase to PCho while GPC and PE are formed as the products of PtdCho and PtdEtn catabolism by hydrolysis mediated by phospholipases. Specifically, an increase in PCho has been reported to be associated with the malignant proliferation by *ex vivo* and *in vitro* NMR studies.^43,44^ Several studies have also documented increased activity of enzymes like choline kinase,^45^ phospholipase A2,^46^ expression of phospholipase C^45^ as well as the upregulation of choline transporters in malignant lesions. These underlying molecular and biochemical processes were suggested as the basis of increased tCho seen in MRS of breast cancer. Thus, increased tCho was thought to be related to increased membrane synthesis and that it may serve as a biomarker for malignant activity and viability of cells. Interested readers may refer to a more detailed review on choline metabolism associated with malignant transformation.^47^

## Methodological aspects

### Acquisition and processing

This section briefly describes some important technical aspects related to acquisition of ^1^H MRS. Till date, most *in vivo* breast ^1^H MRS studies have been performed at 1.5 T;^7–13,22–30^ however, few studies demonstrated its feasibility at higher magnetic fields like 3,^48–52^ 4^31,32^ and 7 T.^33^ MRS at high fields are expected to provide detection of more number of metabolites and possibility of evaluation of small-sized lesions due to increased sensitivity and spectral resolution. The quality of *in vivo* ^1^H MR spectrum depends not only on the strength of magnetic field of scanner but also on the type of breast coil used. Recent developments include the use of multichannel phased array breast coils; however, significant variations were seen in the signal detection from these coils.^53^ Additionally, the use of parallel imaging enables increased signal-to-noise ratio (SNR). Also, the approach based on integrated parallel reception, excitation and shimming has been proposed for brain and abdominal imaging, may have potential in breast imaging.^54^ Development of multichannel transmit combined with multichannel receive array systems have also been reported for breast imaging.^55–57^ Kim et al have developed an RF coil system with eight channel transmit only array that works in conjunction with an eight channel receive only insert and demonstrated its use at 3 and 7 T.^58^

Localized image-guided spectroscopy is used to acquire the MR spectrum from selected region of interest (referred as region of interest or voxel) using either single-voxel (SV) or multivoxel [referred as chemical shift imaging (CSI) or MR spectroscopic imaging (MRSI)] methods. Prior to spectroscopy, DCEMRI is performed for visualization of lesion and positioning of voxel for MRS. Conventional MRI is also useful in large size lesions. It is important that MRS signal is acquired only from the selected voxel and localization techniques play an important role in acquisition. Stimulated echo acquisition mode (STEAM)^59^ and point resolved spectroscopy (PRESS)^60^ are the two widely used pulse sequences that provide good localization for both SV and also for multivoxel, MRS.^61,62^ Another technique, LASER has also been developed for localization of voxel in breast MRS.^63^ The variants of LASER, like semi-LASER, that uses the adiabatic selective refocusing has also been developed for volume localization.^63^ FOCI-LASER pulse sequence provides better detection of lactate signal in tumors and significant suppression of chemical shift artifacts, however, these developments are yet to be implemented in breast MRS.^64^

Breast is composed of fibroglandular and adipose tissues and tissue composition gets altered during malignant transformation. The information on lipid profile and water content of breast tissues is obtained from unsuppressed spectrum, while, suppression of huge water and lipid peaks are essential for detection of tCho signal. A voxel of appropriate size should be carefully positioned well within the tumor avoiding necrotic and fatty areas as these will affect the sensitivity of detection of tCho peak. Further, suppression of huge water and lipid peaks are essential for detection of tCho signal. Water suppression techniques were used in many earlier studies; while recent developments include suppression of both the water and the fat peaks using pulse sequences like MEGA which improves the detection of tCho.^31^ Another method, echo-time averaging also reduces the side-band artifacts of large fat peak.^65^

Good homogeneous magnetic field is mandatory for better diagnostic performance of breast MRS. Therefore, both global and voxel level shimming of the magnetic field should be performed to achieve good field homogeneity for efficient water and lipid suppression. Narrow line width of water and lipid peaks are considered as indicator of good field homogeneity. In our laboratory at 1.5 T, a line-width typically of 10–25 Hz for the lipid peak in normal breast tissues and a line-width of 5–20 Hz for the water peak in patients with breast tumors facilitated good quality ^1^H MR spectrum.^66^ At 3 T, a line-width of 25–30 Hz is generally obtained for the water in SVS. For MRSI experiments, usually for water resonance, a line-width of around 20 Hz is achieved in our laboratory at 1.5 T.^37,66^

Further it is important to acquire the ^1^H MRS of breast using an appropriate echo time. The optimized echo times used are ≥100 ms in various studies.^7–33^ Though at long TE, signal intensity would be less but it provides advantage of reduced lipid side-bands and improved visibility of tCho signal in breast MRS.^7,9^ Including both MRI and ^1^H MRS, the total scan time ranges between 45 and 60 min. To obtain the information on lipid profile of breast tissues unsuppressed spectrum is used.

Following acquisition of the time domain data, processing of the data is performed usually with the software provided by the manufacturer. Post-processing of the acquired FID involves several steps to compensate for the artifacts introduced during acquisition. These include apodization of the time domain data, *i.e*. multiplication by line broadening factor, zero filling followed by Fourier transformation, phase and baseline corrections to improve the quality of the spectrum.^67^ Further, eddy currents produced due to rapid alterations of the gradient magnetic field produce time-dependent shifts in the resonance frequency which leads to the distortion of the spectrum after Fourier transformation. Water signal collected without water suppression is used for frequency corrections induced by eddy currents.^67^ Need for careful referencing of ^1^H MRS spectrum for identification of tCho signal has also been suggested.^67^ Chemical shifts are referenced to water as internal standard at 4.7 ppm.

### Analysis of ^1^H MR spectrum

**Figure 1.**
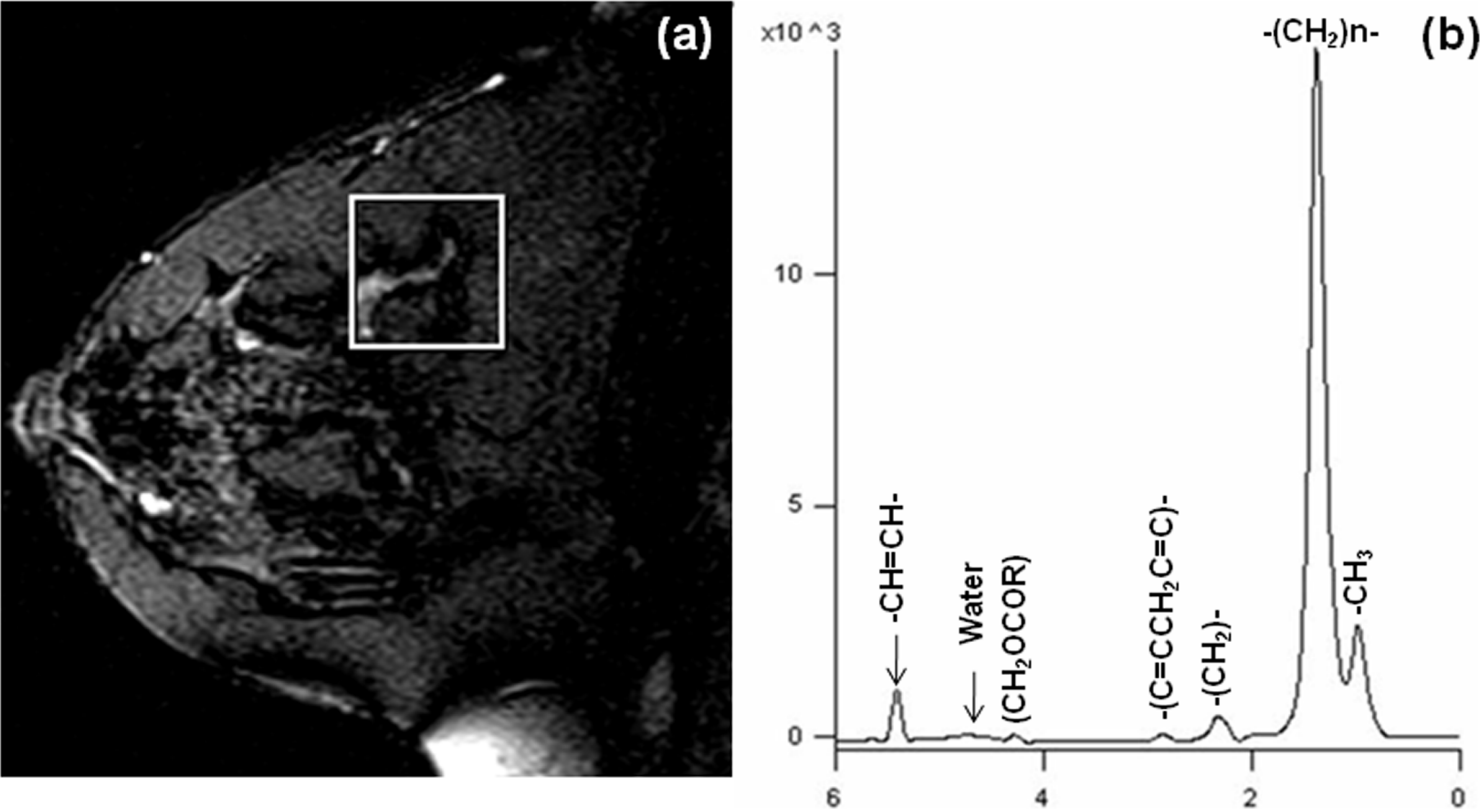
(a) *T*_2_ weighted MR image from the normal breast of a volunteer (35 years old) showing the voxel position from which the ^1^H MR *in vivo* spectrum (b) was obtained without water and lipid suppression (Reprinted from reference 66 with permission from John Wiley & Sons Inc.).

Figure 1a shows the normal breast *T_2_* weighted MR image of a 35-year-old healthy female volunteer while (b) shows the *in vivo* ^1^H MR spectrum acquired without water and fat suppression from the volume of interest (VOI) shown in (a). Normal breast tissues showed a predominant peak at 1.33 ppm due to methylene [-(CH2)_n_] protons of lipids and a peak at 4.7 ppm due to water. The integrals of water and fat peaks are determined and used to calculate various parameters like water to fat ratio, fat fraction and water fraction.^11,15,28^

**Figure 2.**
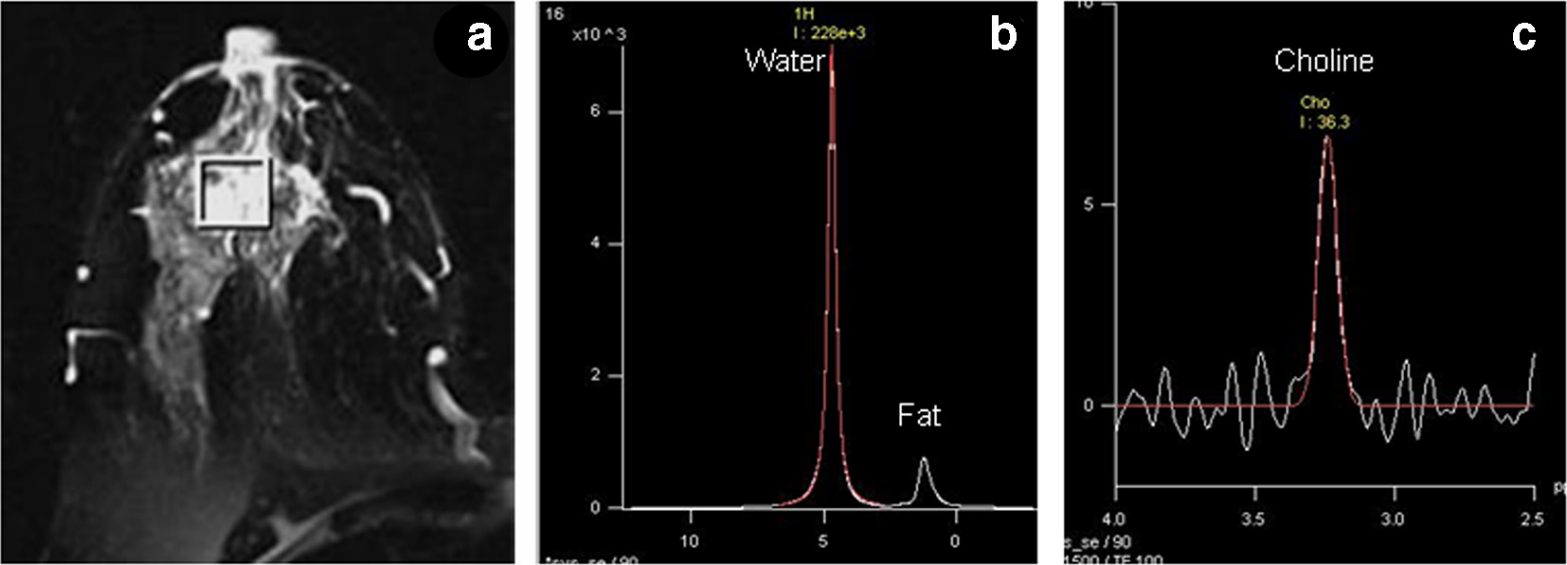
(a) *T_2_* weighted MR image of a patient suffering from locally advanced breast cancer while (b) shows the *in vivo* ^1^H MR spectrum acquired without water and fat suppression from the VOI shown in (a). (c) MR spectrum obtained from the same voxel with water + fat suppression. VOI, volume of interest.

Figure 2a shows the *T_2_* weighted MR image of a patient suffering from locally advanced breast cancer (LABC) while (b) shows the *in vivo* ^1^H MR spectrum acquired without water and fat suppression from the VOI shown in (a). Figure 2c is the MR spectrum obtained from the same voxel with water + fat suppression. As stated earlier tCho peak is detected using water and fat-suppressed spectrum. Following three approaches, namely, qualitative, semi-quantitative and quantitative, have been used for analysis of tCho signal.

### Qualitative approach

The assessment of breast malignancy is subjective in qualitative approach, which is based on the visualization of tCho peak in water suppressed or water + lipid suppressed ^1^H MR spectrum of breast lesion and those with the presence of tCho peak were categorized as positive for malignancy. Several studies reported the potential of breast MRS in increasing the specificity of breast MRI using this approach.^8–13,22^

### Semi-quantitative approach

In the semi-quantitative approach, SNR of tCho (ChoSNR) signal is measured either from SVS or from MRSI. The peak of tCho observed around 3.2 ppm is used to measure the signal amplitude while the spectral region (*e.g.* >9 or<0 ppm) where no signal is expected, is used for the measurement of noise amplitude and ChoSNR is calculated using following formula:

ChoSNR = amplitude of Cho resonance/RMS amplitude of noise

The ChoSNR value **≥**2.0 was considered indicative of malignancy.^37,68^ In a study from our laboratory, the mean ChoSNR for malignant lesions from patients with LABC was 7.9 ± 3.9 which showed a decrease following chemotherapy.^37^ Noise amplitude is affected by several factors like field homogeneity, patient movement, coil loading etc. limiting the utility of this approach. In another semi-quantitative approach, integral value of tCho peak was calculated as area under the peak.^69^ Since tCho integral was presented in arbitrary units without any reference; the data cannot be compared among various sites.

### Quantitative approach

Two types of referencing methods were used for absolute quantification of tCho, namely (a) external, and (b) internal referencing. In external referencing method, the signal intensity of tCho from the VOI in the lesion was compared to that obtained from phosphocholine phantom to estimate the concentration. Roebuck et al calculated tCho concentration in 7/10 malignant lesions in the range of 0.7–2.1 mM in malignant tumors using a 1 mmol l^−1^ choline solution containing phantom as external reference.^7^ On the basis of reference measurements, it was reported that the least detectable tCho concentration was 0.2 mmol l^−1^. Bakken et al used this method in a single patient.^25^ Recently, Mizukoshi et al reported a mean concentration of 1.13 mmol/kg for malignant lesions while a value of 0.43 mmol/kg for benign lesions, using external referencing approach.^70^

Internal referencing approach uses internal tissue water from the same VOI as a reference.^31^ Two ^1^H MR spectra, unsuppressed and water suppressed, are acquired from the same VOI. By comparing the means of a ratio of the tCho integral to the unsuppressed water integral, the concentration of tCho was calculated. This approach offers the advantage that separate calibration experiment is not required like in external referencing method and is also relatively easy to implement. Additionally, several factors such as receiver coil sensitivity, B_0_ shim effects, partial volume effect and radiofrequency transmission efficiency are intrinsically taken into account.^31^ However, variations in water content and its *T*_2_ relaxation rate may affect the quantitation. The concentration of tCho was in the range of 0.8–16.1 mmol/kg for the malignant breast lesions (*n* = 151) and 0.04–2.70 mmol/kg for the benign lesions at 1.5 T using internal referencing approach.^23^ Table 1 presents the concentration of tCho using external and internal referencing method at various magnetic fields.^23–33,70^

**Table 1.** Semi-quantitative & quantitative estimation of tCho in breast lesions in various studies and diagnostic performance of ^1^H MRS by meta-analysis.

**Magnetic field (B_0_**)	**Method** **used**	**Malignant lesions**	**Benign lesions**	**Sensitivity;** **specificity**	**Reference**
**Semi-quantitative assessment**					
**Cho SNR (mean ± SD)/median (range**)					
1.5 T	MRSI	5.7 ± 1.4 (*n* = 5)	2.03 ± 0.3 (*n* = 4)	ND	Jacobs et al.^71^
1.5 T	MRSI	5.9 ± 3.4 (*n* = 27)	2.80 ± 0.8 (*n* = 9)	81%; 78%	Baek et al.^72^
1.5 T	SVS	5.4 (*n* = 19)	No Cho seen (*n* = 16)	100%; 100%	Kim et al.^73^
1.5 T	SVS	2.63 ± 0.16 (*n* = 13)	1.09 ± 0.05 (*n* = 11)	92%; 100%	Lipnick et al.^74^
1.5 T	MRSI	7.1 ± 3.9 (*n* = 30)	ND	ND	Danishad et al.^37^
3.0 T	MRSI	5.7 (0–56.1) (*n* = 32)	2.0 (0–13.6) (*n* = 12)	97%;84%	Gruber et al.^48^
3.0 T	SVS	2.0–29.9 (range) (*n* = 87)	5.4–30.3 (range) (*n* = 28)	89%; 89%	Montemezzi et al^49^
**tCho integral (mean ± SD)/median (range**)					
1.5 T	SVS	2.7 ± 4.2 (*n* = 19)	0.3 ± 0.4 (*n* = 26)	84.2%; 88.5%	Sardanelli et al.^69^
3.0 T	SVS	2.04 ± 2.00 (*n* = 42)	0.09 ± 0.32 (*n* = 15)	95.2%; 93.3%	Suppiah et al.^50^
3.0 T	SVS	122.2 ± 124.5 (*n* = 25)	29.7 ± 47.2 (*n* = 26)	96.0%; 65.4%	Ramazan et al.^51^
3.0 T	SVS	0.2–51 (range) (*n* = 75)	0–11 (range) (*n* = 63)	86.7%; 63.5%	Aribal et al.^52^
**Quantitative assessment of tCho concentration (mmol/Kg**)					
1.5 T	SVS	0.7–2.1 (*n* = 10)	5.8 (*n* = 1)	70%; 86%	Roebuck et al.^7^
1.5 T	SVS	2.0 (*n* = 1)	ND	ND	Bakken et al.^25^
1.5 T	SVS	0.8–21.2 (*n* = 32)	ND	ND	Baik et al.^26^
1.5 T	MRSI	4.1 and 4.6 (*n* = 2)	ND	ND	Sijens et al.^27^
1.5 T	SVS	0.0–47.1 (*n* = 57)	0.0–1.4 (*n* = 31)	75–96% ; 93–100%	Thakur et al.^28^
1.5 T	MRSI	1.7–11.8 (*n* = 15)	0.4–1.5 (*n* = 11)	100%;100%	Dorrius et al.^29^
1.5 T	SVS	0.8–16.1 (*n* = 151)	0.04–2.70 (*n* = 38)	76%; 75%	Sah et al.^23^
1.5 T	SVS	0.08–9.9 (*n* = 62)	ND	ND	Chen et al.^30^
1.5 T	SVS	1.13 ± 0.92 (*n* = 169)	0.43 ± 0.42 (*n* = 39)	68.1%; 79.4%	Mizukoshi et al.^70^
4.0 T	SVS	0.4–10.0 (*n* = 86)	ND	46%; 94%	Bolan et al.^31^
4.0 T	SVS	0.0–8.5 (*n* = 35)	0.0–1.40 (*n* = 20)	ND	Meisamy et al.^32^
7 T	MRSI	0.5–4.2 (*n* = 2)	ND	ND	Klomp et al.^33^
**Diagnostic performance of ^1^H MRS as evaluated by meta-analysis and systematic review**					
Number of studies	No. of lesions	Parameters evaluated	Pooled sensitivity	Pooled specificity	
19	Malignant = 773; Benign = 452	Visual/tCho SNR/tCho integral/tCho conc.	73% (CI 64–82%)	88% (CI 85–91%)	Baltzer & Dietzel^16^
18	Malignant = 750; Benign = 419	Visual/tCho SNR/tCho integral/tCho conc.	71% (CI 68–74%)	85% (CI 81–88%)	Cen & Xu^17^
10	Malignant = 480; Benign = 312	tChoSNR	74% (CI 69–77%)	76% (CI 71–81%)	Wang et al.^18^
16	Malignant = 661; Benign = 388	Visual/tCho SNR/tCho integral/tCho conc. Only from post contrast studies	74% (CI 70–77%)	78% (CI 73–82%)	Tan et al.^19^

Cho SNR, total choline SNR; MRSI, magnetic resonance imaging; ND, not determined; SD, standard deviation; SNR, signal-to-noise ratio; SVS, single voxel spectroscopy; tCho, total choline.

## Identification of biomarkers and their diagnostic significance

In breast ^1^H MRS studies, the important parameters that are determined include; (a) water-to-fat ratio (W–F), fat fraction, water fraction from the unsuppressed spectrum, and (b) tCho from water or water + fat-suppressed spectrum. The potential of these parameters was evaluated in understanding the altered lipid and choline metabolisms associated with breast cancer and their role in the diagnosis^7–33,48–52,75^ and in assessing the tumor response to various therapeutics.^8,11,37,39,40,76,77^

### Lipid metabolism and its role in the diagnosis of breast cancer

Malignant transformation is associated with the alterations in lipid metabolism which is manifested as altered water and lipid composition in breast tissues. Several *in vivo* ^1^H MRS studies have reported these metabolic changes by monitoring the spectral characteristics of water and fat resonances and the usefulness of various parameters like W–F ratio, fat fraction and water fraction in characterizing breast malignancy.^76–80^ The predominance of fat characterizes the normal breast tissue (Figure 1b) while tumor spectrum show prominent water peak indicating that malignant tumors are characterized by high water content (Figure 2b).^6^ Studies have compared the W–F ratio of malignant and benign lesions and also evaluated its potential in therapeutic monitoring.^12,76,77^ *In vivo* localized correlated two-dimensional MRS also reported the W–F ratio using two-dimensional cross-peak volumes and suggested the association between tumor lipid content with its development and progression.^81^ These earlier studies concluded limited diagnostic utility of W–F ratio as a diagnostic biomarker due to significant overlap seen in the W–F ratio of benign and malignant breast lesions. Additionally, the variability of water content in relation to glandular and fatty tissue composition of breast, its association with age and other physiological factors like menstrual cycle were reported as significant factors that would limit its diagnostic utility.^14^ It was reported that menstrual cycle influences the W–F value in the para-areolar region of the normal breast tissue and thus location of the tumor within the breast as well as the time of menstruation should be carefully taken into consideration for assessment of breast pathology using W–F values.^82^

Wang et al, in their *in vivo* ^1^H MRS study of normal healthy volunteers (including females without family history of breast cancer, females with at least one affected first degree relative and contralateral breast of newly diagnosed cancer) demonstrated the association of water and lipid composition with the risk factors for breast cancer. Breast density was found to be positively correlated with the water fraction in all the groups.^80^

Recently, Agarwal et al reported lower fat fraction in malignant compared to benign lesions and normal breast tissue of healthy volunteers.^78^ The sensitivity and specificity of fat fraction was 76 and 74.5%, respectively to differentiate malignant and benign lesions. Lipid metabolism was investigated at 7 T using ^1^H MRS which facilitated quantification of six lipid metabolites and documented differences in the fatty acid composition between malignant and benign lesions and luminal A/B *v* *s* other molecular subtypes of breast cancer.^75^

### Role of tCho in the diagnosis of breast cancer

The potential of tCho as a non-invasive biomarker in differentiating malignant from benign breast lesions have been evaluated.^7–33^ An intense peak of tCho with high concentration was seen in the MR spectrum obtained with water + fat suppression for malignant lesions (Figure 2c). Table 1 presents the semi-quantitative and quantitative estimates of tCho and its sensitivity and specificity obtained from various studies. It may be noticed that the lower concentration of tCho was reported in benign lesions in various ^1^H MRS studies (Table 1).

An earlier meta-analysis of the data of initial five studies that used qualitative approach gave a combined sensitivity and specificity of ^1^H MRS as 83 and 85%, respectively in distinguishing malignant from benign breast lesions.^13^ In younger patients (≤40 years of age), higher sensitivity (100%) and specificity (89%–100%) was documented in the subgroup analysis.^8–10^ Following these studies, several review articles^83–86^ presented the sensitivity and the specificity obtained from various MRS studies and several articles on meta-analysis of breast MRS data were published.^16–20^ Baltzer and Dietzel in 2013 included 19 breast MRS studies in the meta-analysis and reported a pooled sensitivity and a specificity of 73 and 88%, respectively.^16^ This meta-analysis combined the data of all the studies based on qualitative, semi-quantitative and quantitative assessments. In 2014, Cen and Xu reported a meta-analysis of 18 SVS breast MRS studies that included 750 malignant and 419 benign lesions.^17^ The pooled sensitivity and specificity of MRS in their analysis was 71 and 85%, respectively, and standardization of the acquisition protocol for MRS across the multicenter trials was recommended.^17^

### Factors affecting diagnostic performance of tCho

The lesion size plays an important role in the delectability of tCho signal. In a study by Tozaki et al^87^ ^1^H MRS was performed prior to biopsy on BIRADS 4 and 5 category lesions (*n* = 171). The sensitivity was only 44% when all lesions were included in the analysis, while it improved to 82% (28/34) when mass lesions greater than 1.5 cm were only included indicating that lesion size plays an important role in the detection of tCho.^13^ However, false negative findings were seen even for relatively large invasive cancers, in addition to infiltrative ductal carcinoma (IDC).^88^ Further, the diagnostic performance of breast MRS is reported to be lower in non-mass lesions.^16^ Subgroup analysis of mass and non-mass lesions from six studies showed that pooled sensitivities were 68 and 62% while specificities were 88 and 69%, respectively.^16^

Furthermore, tCho detection rate has been found to be associated with the histology of breast cancer. Bartella et al documented a high sensitivity of 100% and a specificity of 85% for enhancing non-mass lesions.^89^ This was reported to be due to the differences in the histology of breast cancer. The number of patients with DCIS type lesion were less in the study by Bartella (17%)^89^ compared to the study of Tozaki et al (89%).^87^ The sensitivity of breast MRS also decreased due to false negative findings in various histological types of breast cancers like medulllary carcinoma,^9,90^ mucinous carcinoma^87^ and apocrine carcinoma.^87^ Among the benign lesions, false positive findings were mostly reported in fibroadenoma,^8,9,87,89^ tubular adenoma,^7,10^ intraductal papilloma,^87^ atypical ductal hyperplasia,^69,83^ inflammatory lesions with atypia^83^ and mastopathy.^8,10,69,87^

The results of the various studies (Table 1) suggested the need for optimization of cut off values of semi-quantitaive^37,48–52,69,71–74^ and quantitative estimates^7,23,25–33,52,70^ of tCho to classify malignancy across the various research centers. For example, Bartella et al^68^ reported a cut-off ChoSNR value as ≥2 while Baek et al^72^ used a cut-off ChoSNR value of >3.2 to differentiate malignant from benign lesions that resulted in 81% sensitivity, 78% specificity and 81% accuracy. Wang et al reported a pooled sensitivity and a specificity of ChoSNR as 74 and 76%, respectively, based on meta-analysis of 7 ^1^H MRS studies which included 371 malignant and 239 benign lesions.^18^ It was reported that semiquantitative parameter, ChoSNR is easily measureable and has similar diagnostic performance to the quantitative estimates of tCho. Further tChoSNR ≥2 as cutoff for malignancy provided better diagnostic accuracy.^18^

Similarly, there is a need to arrive at a cut-off value of tCho concentration for differentiation of malignancy. Till date, several studies have reported the concentration of tCho in large number of malignant and benign lesions and the reported sensitivity and specificity of diagnosis was in the range of 46–100% and 75–100%, respectively^7,23,25–33,49–52,71–74^ (Table 1). In a study from our laboratory, tCho concentration was determined in LABC (*n* = 120) and early breast cancer patients (*n* = 31) at 1.5T. The tCho concentration was in the range of 1.7–11.8 mmol/Kg for LABC patients while it ranged from 0.8 to 16.1 mmol/kg in early breast cancer patients.^23^ Accordingly, a cut-off value for tCho was calculated as 2.54 mmol/kg for the differentiation of malignant from benign breast tissues. A value of 1.45 mmol/kg was observed as the cut-off value for malignant *vs* normal; and between benign and normal breast tissues the value was 0.82 mmol/kg.^23^ This disparity may be due to lesion size and various technical limitations related to field homogeneity at 4.0 T^38^.

**tCho in lactating and normal breast tissues**

The observation of tCho is not restricted to malignant and benign breast lesions. It is seen in normal breast tissues of healthy volunteers (though in lesser concentration) and in the normal breast tissues of lactating females. This raises the question of the diagnostic ability of ^1^H MRS.^8,11,22^ Recently, we reported tCho concentration in normal breast tissues of healthy lactating females volunteers (*n* = 12) and compared it with malignant lesions.^24^ The concentration was 3.51 ± 1.72 mmol/kg in malignant lesions which was similar to that calculated for lactating females (3.52 ± 1.70 mmol/kg). The normal breast MR spectrum of 10/12 healthy lactating females volunteers showed a lactose peak in addition to tCho resonance, which was unique in lactating females and not observed in breast cancer patients. It was reported that mammalian milk contains free lactose which constitutes more than 80% of the total carbohydrate content and is important for lactogenesis.^91^ Presence of lactose peak was also reported by Stanwell et al using *in vivo* ^1^H MRS in lactating breast tissue which was attributed to increased metabolic activity of epithelial cells for apocrine and merocrine secretions.^67^ Further it was reported that in lactating females, the major constituent of tCho peak seen around 3.2 ppm was GPC, while in breast cancer it was PCho through careful referencing.^67^

Further, the observation of higher apparent diffusion coefficient in addition to lactose was unique feature of healthy lactating females volunteers that differentiated them from malignant lesions.^24^

### Association of tCho with molecular markers

Tozaki and Hoshi^88^ reported correlation of tCho levels with nuclear grade, estrogen receptor status and triple negative status. Recently, we reported a significantly lower tCho concentration (*p* < 0.05) in patients with triple negative receptor status compared to those with triple positive and non-triple negative status.^23^ These findings indicated the heterogeneity of breast malignancy and the complex nature of molecular mechanism of cell. The association of tCho with the Wnt/β-catenin pathway in breast cancer was recently studied by us.^34^ A positive correlation was seen between tCho and cytosolic and nuclear expressions of β-catenin and cyclin D1, in malignant tissues. Progesterone receptor negative patients had higher cytosolic β-catenin expression than progesterone receptor positive patients.^34^ Baio et al^92^ reported a correlation between choline and the expression of calcium-sensing receptors, which indicated its role in the synthesis of choline in breast malignancy.

### High-field MRS

Increased sensitivity and spectral resolution of^ 1^H MRS are observed with the increased field strength. Further, it is possible to use reduced voxel size which would facilitate the possibility of evaluating small sized lesions^93,94^ and detection of more number of metabolites other than Cho. However, lipid side-bands and respiratory induced shifts will increase at high frequency. Also, increased magnetic susceptibility needs to be minimized by B_0_ shimming.^38^ The MRS at 4 T evaluated the feasibility of using smaller voxel size of 1–2 ml in breast cancer patients and reported the error in concentration calculation in voxels smaller than 1 ml size.^31^ In addition, MRS at high fields has several challenges like difficulty in B_0_ shimming and B_1_ in-homogeneities due to complexities of coil design. Further, relaxation rates are higher that require use of long repetition time and echo time values.

Recently, few ^1^H MRS studies at 3 T have been reported in breast cancer patients.^48–52^ Montemezzi et al^49^ evaluated patients with BI-RADS 4–5 lesions at 3 T using SVS. They reported reliable spectra in 115/127 lesions, however, a tCho peak with SNR ≥2 was detected only in 66 malignant and 3 benign lesions. Vassiou et al^95^ reported the ^1^H SVS of 15 malignant and 11 benign breast lesions. The qualitative assessment based on tCho observation in MRS showed 80% sensitivity and 81.8% specificity with an accuracy of 80.7%. Aribal et al^52^ evaluated the diagnostic accuracy of multi parametric breast MR including DCEMRI, diffusion MRI and ^1^H MRS in differentiating malignant (*n* = 75) and benign (*n* = 63) lesions at 3 T. They reported a cut-off value of tCho integral as 3.2 with sensitivity and a specificity of 86.67 and 63.49%, respectively. The study concluded that combination of DCEMRI, diffusion MRI and ^1^H MRS reduced the diagnostic accuracy of breast MRI.^52^ Ramazan et al^51^ detected tCho peak in 24/25 malignant and 9/26 benign lesions by ^1^H MRS at 3.0T. Choline peak was not detected in 1 case of DCIS. They reported a sensitivity of 96%; however, the specificity was only 65%. Kousi et al^96^ detected tCho in 11/14 malignant lesions at 3 T while no tCho signal was detected in 12/13 benign lesions. Though several studies used high field MRS, still the benefits expected in comparison to MRS studies performed at 1.5 T are yet to be realized with optimization of MRS procedure in a clinical setting.

### Combined use of W-F, tCho and and lipid estimates in breast cancer differentiation

In a recent study Clauser et al^79^ evaluated the SNR of tCho, olefinic acids (5.34 ppm), and ratio of water to methylene peak (1.33 ppm) and demonstrated the use of these three variables in the differentiation of malignant and benign lesions. Using the classification algorithm χ^2^–automatic–interaction–detection, these three variables was found to be useful in avoiding false-positive diagnosis in benign lesions. Thus, it was suggested that evaluation of multiple spectral regions can reduce the false-positive findings and increase the diagnostic performance of ^1^H-MRS.^79^ Thakur et al^28^ compared tCho and W/F ratios of various subtypes of malignant and benign lesions along with the normal breast parenchyma. Diagnostic usefulness of both these parameters was demonstrated to improve when used in combination. Additionally, W–F ratio differentiated infiltrative ductal carcinoma and ILC lesions while tCho levels were similar for these two subtypes of breast cancer.^28^

### ^1^H MRS in evaluating therapeutic response

In addition to the diagnostic ability of ^1^H MRS, number of studies has demonstrated its potential in monitoring therapeutic response of patients undergoing neoadjuvant chemotherapy (NACT). W-F ratio reduced following chemotherapy in LABC patients indicating its utility as a noninvasive biomarker of positive outcome of therapy.^2,76,77^ W–F ratio showed 100% sensitivity and negative-predictive value in accurately predicting non-responders.^77^ The W–F ratio showed limitation in the characterization of diffuse breast cancers and lobular carcinoma.^77^

Use of tCho for monitoring response was first demonstrated by Kvistad et al in a single patient.^8^ Later, our group reported the role of tCho in monitoring the chemotherapeutic effects in 14 LABC patients after the third or sixth cycle of NACT.^11^ Before therapy 10/14 cases showed tCho, while after therapy out of these 10 cases, tCho signal was not seen in seven indicating a positive response to NACT that also correlated with the clinical and histology response.^11^ tCho integral and ^18^F-fluorodeoxyglucose uptake values were also shown to predict the chemotherapeutic response in seven breast cancer patients.^97^ Our group also demonstrated that both tumor size and ChoSNR reduced in responders after therapy while there was no significant change in the values of these two parameters in non-responders, after NACT.^37^

**Figure 3.**
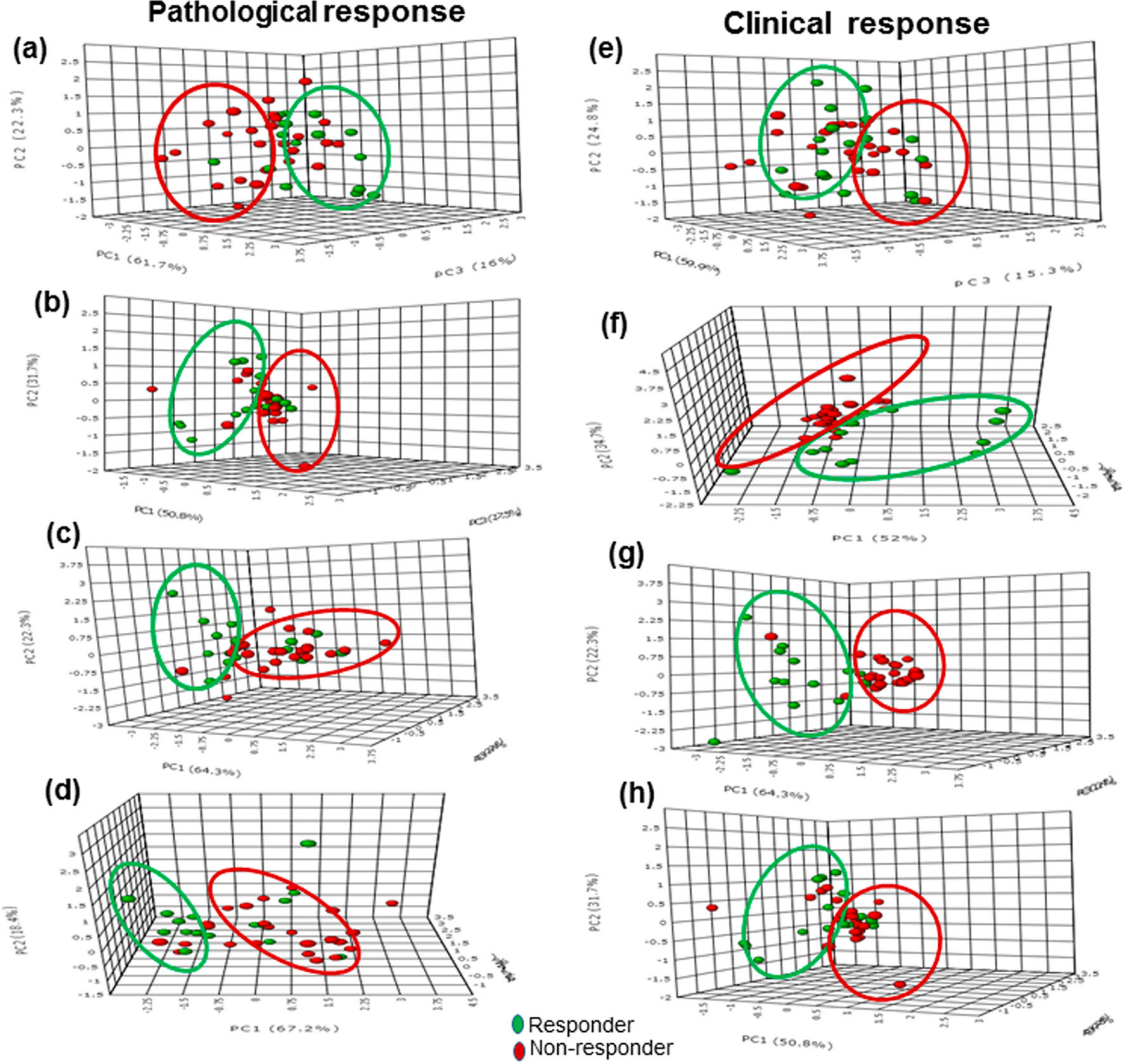
The 3D score plot (PC1-PC3) of PCA analysis of multiparametric data (volume, ADC and tCho) in pathological responders and non-responders at pre-therapy-Tp0 (a) after I NACT-Tp1 (b), II NACT-Tp2 (c), and after III NACT-Tp3 (d), while (e–h) show the 3D score plot for clinical response (Figure as originally published in reference 39: Uma Sharma, Khushbu Agarwal, Rani G. Sah, Rajinder Parshad, Vurthaluru Seenu, Sandeep Mathur, Siddhartha D. Gupta and Naranamangalam R. Jagannathan (2018). Front. Oncol. 15 August 2018 doi: 10.3389/fonc.2018.00319). 3D,three-dimensional; NACT,neoadjuvant chemotherapy; PCA, principal component analysis.

Meisamy et al demonstrated a significant change in tCho concentration that was evident as early as 24 h of treatment in clinical responders at 4 T.^32^ Later, another study at 4 T from the same group reported decreased tCho concentration in 75% responders while no change or an increase in 92% non-responders after Day 1 of chemotherapy.^38^ Both tCho concentration and the tumor size showed changes in patients with complete pathological response, after one or two cycles.^98^ Recently, our group reported the potential of multiparametric approach using tCho, apparent diffusion coefficient and tumor volume in predicting both pathological and clinical responses in 42 LABC patients undergoing NACT (Figure 3).^39^ Significant changes were seen as early as first NACT in both tCho and ADC while tumor volume reduced after second cycle of therapy in both pathological and clinical responders.^39^ Recently, Leong et al reviewed the various studies that used MRS and DWI in evaluating the therapeutic response in breast cancer patients and discussed the strengths and limitations of both the techniques.^40^

## SUMMARY, LIMITATIONS & FUTURE DIRECTIONS

This review briefly discussed the methodology, technical details and the applications of *in vivo* breast ^1^H MRS. The various MRS studies carried out at 1.5 T and at higher magnetic fields demonstrated its potential in the diagnosis and the assessment of therapeutic response of breast tumors. The diagnostic ability of breast MRS also decreased due to false-negative findings in various histological types of breast cancers like medulllary carcinoma, mucinous carcinoma, apocrine carcinoma and angiosarcoma and also false positive findings in benign lesions. These reports suggested the need for evaluating more number of various histological types of breast lesions using MRS. Association of tCho with molecular/hormonal markers facilitates a better understanding of the heterogeneity of breast lesions. MRS has also shown its potential in monitoring the early tumor response to therapy, an important aspect in the management of breast cancer patients. Despite many years of development in breast coil design, use of high magnetic field strengths for MRS, post-processing algorithms etc., it still remains a challenge to visualize and quantify tCho in small-sized tumors in a routine manner and to integrate the technique in a clinical setting. Also, most high-field studies reported qualitative findings; however, acquisition, processing and quantification procedures of MRS at high-fields require further improvements in detecting tCho signal and other metabolites. Future research should focus on the use of advanced acquisition methods like use of parallel imaging, faster shimming algorithms, development of coils which provide better comfort for patients and easy quantitative methods for the estimation of the tCho concentration. Additionally, to achieve the integration of breast MRS in routine clinical setting, multicenter studies are necessary.
